# A narrative review of the ethnomedicinal usage of *Cannabis sativa* Linnaeus as traditional phytomedicine by folk medicine practitioners of Bangladesh

**DOI:** 10.1186/s42238-021-00063-3

**Published:** 2021-03-19

**Authors:** Shahriar S. M. Shakil, Matt Gowan, Kerry Hughes, Md. Nur Kabidul Azam, Md. Nasir Ahmed

**Affiliations:** 1 Department of Pure and Applied Biochemistry, Lund University, Lund, Sweden; 2 The Canadian College of Naturopathic Medicine, Toronto, Ontario Canada; 3 Ethnopharm LLC, California City, USA; 4 Department of Genetic Engineering & Biotechnology, Jashore University of Science & Technology, Jashore, Bangladesh; 5 Biotechnology & Natural Medicine Division, TechB Nutrigenomics, Dhaka, Bangladesh

**Keywords:** Cannabis, Cannabinoids, Ethnomedicine, Phytomedicine, Traditional medicine, Folk medicine practitioner, Bangladesh

## Abstract

**Background:**

There is a worldwide interest in the use of *Cannabis sativa* for biomedicine purposes. Cannabis has ethnomedicinal usage as a natural medicine in Bangladesh and cultivated during the British Empire period for revenues.

**Objective:**

Folk medicine practitioners (FMPs) from different districts of Bangladesh have been using *Cannabis sativa*, but until now there have not been any compiled studies particularly regarding this practice. Hence, this review is an effort to retrieve the traditional usage of *Cannabis sativa* as a phytomedicine from published ethnomedicinal studies.

**Methods and materials:**

Information was searched by using the search terms “ethnomedicinal *Cannabis sativa* and Bangladesh”; “Bangladesh cannabaceae and ethnomedicinal survey”; “ganja, bhang and folk medicine Bangladesh”; “tetrahydrocannabinol (THC), cannabinoid and therapeutic, clinical trial”; and “cannabis and pharmacological/biological” and retrieved from ethnobotanical articles available on PubMed, Scopus, Science Direct, and Google Scholar databases. A search of the relevant scientific literature also was conducted to assess the efficacy of the ethnomedicinal usage of *Cannabis sativa*.

**Results:**

While reviewing over 200 ethnomedicinal plants’ survey articles, we found that FMPs of Bangladesh from 12 different districts used *Cannabis sativa* to treat cited ailments like sleep-associated problems (*n*=5), neuropsychiatric and CNS problems (*n*=5), and infections and respiratory problems (*n*=5) followed by rheumatism, gastrointestinal, gynecological (*n*=4 each), cancer, sexual, and other ailments including hypertension, headache, itch, increases bile secretion, abortifacient, dandruff, fever, and urinary problems (*n*=1 each). There are a total of 15 formulations identified from the 11 out of 18 ethnomedicinal plant survey reports. The leaf was the main plant part used (53.8%), followed by root (23%), seed (7.7%) and flower, inflorescence, resin, and all parts 3.8% respectively.

**Conclusions:**

Sales and cultivation of Cannabis are illegal at present in Bangladesh, but the use of *Cannabis sativa* as a natural phytomedicine has been practiced traditionally by folk medicine practitioners of Bangladesh for many years and validated through relevant pharmacological justification. Although *Cannabis sativa* possesses ethnomedicinal properties in the folk medicine of Bangladesh, it is, furthermore, needed to conduct biological research to consolidate pharmacological justification about the prospects and challenges of Cannabis and cannabinoids’ use in Bangladesh as safer biomedicine in the future.

## Background

Medicinal plants are useful for healing properties since the ancient world and essential resources for developing biomedical drugs (Šantić et al., 2017, Souza et al., 2018). Ethnomedicine or traditional medicine knowledge usually refers to the medication of any particular culture that examines local ideas and behaviors of how to treat illness and how to stay healthy (Quinlan 2011). In General, ethnomedicinal knowledge is practiced and passed verbally from one generation to the members of the family of the following generation, and traditional medicine practices by utilizing medicinal plants are now being declined because of modernization and destruction of the medicinal plants, reduction of the interest in the younger generation, and switching to other jobs, but such knowledge of traditional medicine has been a way towards the invention of the many new medicines (Faruque et al. 2018, Singh et al. 2020).

Bangladesh, officially the People’s Republic of Bangladesh, is known as a country of cultural, ethnic, and language diversities. To date, this country has 7 divisions, 64 districts, 492 sub-districts, and 87,310 villages. The country possesses enormous resources of medicinal plants and traditional phytomedicine is still practiced throughout the country. Folk medicine practitioners (FMPs) in Bangladesh are commonly known as *Kabiraj* in the mainstream community and there are *kabirajes* in almost every village in Bangladesh. *Kabirajes* are important sources of ethnomedicinal knowledge which they have inherited from their ancestors (Rashid 2017). They enjoy considerable trust and support from their patients thanks to their holistic approach to treatment (Biswas et al. 2011). *Kabirajes* rely mainly on medicinal plants, dispensing medications for several health disorders and diseases including psychosis, cardiovascular disorders, eye infections, malaria, leucorrhea, leprosy, helminthic infections, urinary tract infections, sexually transmitted diseases, snakebite treatment, diabetes, gastrointestinal disorders, tumor, and cancer (Ahmed and Azam 2014, Azam et al. 2014, 2015, 2016, Hasan et al. 2012, 2015, Hossan et al. 2010, Mollik et al. 2009, Rahmatullah et al. 2010a, 2010b, 2010c, 2010h, 2012, 2012a). Each *Kabiraj* normally keeps his or her knowledge of medicinal plants within the family and passes this knowledge through from generation to generation. Over time, this knowledge becomes unique to the *Kabiraj* and his successors (Jahan et al. 2011).

*Cannabis sativa* is perhaps the most famous plant ever discovered by humans and the plant has a rich history with complex metabolic biology and fascinating physiology (Zwenger 2014). *Cannabis sativa* (hereafter referred to as “Cannabis”) is an angiosperm belonging to the Cannabaceae family. For more than 5000 years, *Cannabis sativa*, also known as Marijuana, has been cultivated for fibers and source for seed oil (Lash 2010, Leizer et al. 2000) and for medicinal (Clarke and Merlin 2013, Zuardi 2006) and recreational use (Small 2017) around the world (Burstein 1997, Cota et al. 2003). In Arabic-Islamic medicine, *Cannabis sativa* extract has been used for its multiple curative properties such as diuretic, anti-emetic, anti-epileptic, anti-inflammatory, anti-parasitic, antipyretic, anti-bacteria, anti-tumor, vernucide and vermifuge, dermatic, carminative, and pain-killing properties; the most used part was seeds; and the common methods of preparation were seeds’ oil and juice from the leaves and green seeds’ oil (Lozano 2001). Regardless, the Cannabis leaves alone have the potential to treat quite 25 different types of diseases (Kala et al. 2004), and other parts of the plant including the seeds, flowers, and roots are also being used.

*Cannabis sativa* is a wonder plant with over 500 chemical entities (Radwan et al. 2009) including over 100 phytocannabinoids and 120 terpenes (Calvi et al. 2018), of which less than 30 biosyntheses have been characterized (Booth and Bohlmann 2019). Phytocannabinoids are divided into 10 subclasses, i.e., cannabigerol, cannabichromene, cannabidiol, (−)-*trans*-Δ^9^-tetrahydrocannabinol, cannabicyclol, cannabielsoin, cannabinol, cannabitriol, and miscellaneous (Brenneisen 2007). There has been also a new phytocannabinoid identified which is called Δ9-tetrahydrocannabiphorol (Δ9-THCP) (Citti et al. 2019). Recently, the molecular pharmacology of the seven most phytocannabinoids (namely Δ9-tetrahydrocannabinol, Δ9-tetrahydrocannabivarin, cannabinol, cannabidiol, cannabidivarin, cannabigerol, and cannabichromene) was investigated thoroughly (Turner et al. 2017), and 14 cannabinoids, 47 terpenoids, 3 sterols, and 7 flavonoids have been profiled in Cannabis flowers, leaves, stem barks, and roots for medicinal purposes (Jin et al. 2020). Moreover, the medicinal effects of *Cannabis sativa* are exploited within the treatment of epilepsy, pain, anxiety, depression, sleep disorders, nausea related to cancer treatment, and psychotic conditions, and it has antiglaucoma, antiemetic, antiobesity, and anticancer properties (Andre et al. 2016, Barrales-Cureño et al. 2020, Corroon and Phillips 2018). In addition, the government of Canada recognizes *Cannabis sativa* as an effective treatment for over two dozen conditions (Health Canada 2018). It is noteworthy that the scientific interest in *Cannabis sativa* was renewed in the early 1990s with the outline of cannabinoid receptors and therefore the identification of an endogenous cannabinoid system in the nervous system of humans (Zuardi 2006) wherein the pharmacological mechanisms of Cannabis metabolites would guide advances in therapies and changes in public health approaches (Russo and Marcu 2017).

### History of *Cannabis sativa* cultivation in Bangladesh: usage and law

Cannabis has a long history of production and uses in Bangladesh. The British Empire began producing revenues from the production and trade of Cannabis goods in its South Asian territories in the early nineteenth century. Naogaon, a district of Bangladesh (formerly known as Eastern Bengal), was the single largest cultivation zone of Cannabis in colonial South Asia. The “Naogaon Ganja Cultivators’ Cooperative Society limited” which was registered in 1917, was arguably the most successful cooperative in colonial India. The cooperative society obtained sole monopoly award from the British government and allowed it to control all sales of Cannabis (Chattopadhyaya 2018). They sold Cannabis only to the licensed vendors at a rate that was fixed by the Commissioner of Excise and redistributed profits annually for the benefit of the public. The society also contributed to King George’s Sailors’ Fund and to the local board for improving the roads and bridges, etc. (Report of the Bengal Provincial Banking Enquiry Committee, 1929-30, 1930). During the British colonialism period of the East India Company, Cannabis was marketed in Asia in a variety of formulations using flower and resin with low psychotropic content (named Bhang) and high psychoactive content (named Ganja or Charas) (Bonini et al. 2018). During the mid-twentieth century, many of the novels of a popular Bengali novelist Sarat Chandra Chattopadhyay reveal that smoking Ganja among the elderly people was common and socially accepted (Mahmud 2008). The use of ganja and its consumption for spiritual purposes has been practiced and tolerated in Bangladesh society for thousands of years (Haque 1993). The Muslims, particularly the Sufis of Bangladesh, have a tradition of using ganja for their spiritual purposes (Malek 1999). A leading English daily in Bangladesh reported on a 3-day traditional Ganja festival in 2004 and they published the following:

“Thousands of marijuana or ganja lovers seeking spiritual ecstasy from across the country as well as from India, Nepal, Bhutan, Myanmar and Pakistan have started pouring in the 2,375 year-old Mahastangarh to celebrate a three-day ganja festival beginning today. The annual extravaganza, a crowd-puller, is held every last Thursday of the Bangla month of Boishakh and draws both male and female pot connoisseurs with plaited hair” (Bilu 2004; archive.thedailystar.net/2004/05/13/d40513011111.htm).

In 1971, marijuana became popular as a “natural drug” among the young generation due to the mental trauma of the historic War of Liberation and to avoid post-war suicidal tendencies among the glorious fighters. The renowned musician and author Maqsoodul Haque from the book of *History of Bangladesh Rock, the legacy of Azam Khan* stated the following:

They lived, trained and fought for independence in villages where Marijuana was to a large degree socially acceptable. Its contribution to our Liberation War therefore must be acknowledged, because I personally know of at least a dozen past Mukti Bahini guerrillas who went into battle ‘stoned out of their mind’ to beat back fear and pain. (Haque 2020)

The cultivation and sale of Cannabis is now illegal in Bangladesh; however, as per schedule I of the Narcotics Control Act, 1990, marijuana is a B-Class narcotics and section 9 of the Act has given permission to manufacture, process, import, export, supply, purchase, and sell narcotics for any approved medicine or for undertaking any scientific research which is being done under proper license, being used with the proper permit, and being carried or transported with proper pass (Joy 2017; www.thedailystar.net/law-our-rights/your-advocate-1419271)

In this review, we aimed to document ethnomedicinal usage of *Cannabis sativa* by the FMPs of Bangladesh, retrieved from published ethnomedicinal plants’ survey reports. We also reviewed the relevant scientific literature to consolidate the ethnomedicinal efficacy of *Cannabis sativa* in Bangladesh.

## Methods and materials

The literature search for the ongoing study was conducted several times from August 2013 to March 2020 and updated on 3 December 2020 using the following multiple search terms through electronic sources of scientific databases like PubMed, Scopus, Science Direct, and Google Scholar: “ethnomedicinal *Cannabis sativa* and Bangladesh”; “Bangladesh cannabaceae and ethnomedicinal survey”; “ganja, bhang and folk medicine Bangladesh”; “tetrahydrocannabinol (THC), cannabinoid, and therapeutic, clinical trial”; “cannabis and pharmacological/biological”; etc.

*Cannabis sativa* together with other medicinal plants used as folk medicine has been documented randomly by Bangladeshi researchers from diverse affiliations through ethnomedicinal plant survey methods. Later, the study data have been published in English language by the authors in manuscript form in several journals. We identified these articles and retrieved information on the ethnomedicinal uses of *Cannabis sativa* reported by FMPs of Bangladesh.

### Data analysis

#### Plant part value (PPV)

The percentage of utilized plant parts (root, seed, leaves, flower, fruit, etc.) is calculated according to Gomez-Beloz (2002) as follows:

$$ \mathrm{PPV}\left(\%\right)=\frac{\Sigma \kern0.5em RU\left(\mathrm{plant}\kern0.17em \mathrm{part}\right)}{\Sigma \kern0.5em RU}\times 100 $$

Here, RU is the total number of uses reported of all parts of the plant and RU (plant part) is the sum of uses reported per part of the plant.

## Results

### Ailments treated by the folk medicine practitioners (FMPs) in Bangladesh

**Fig. 1:**
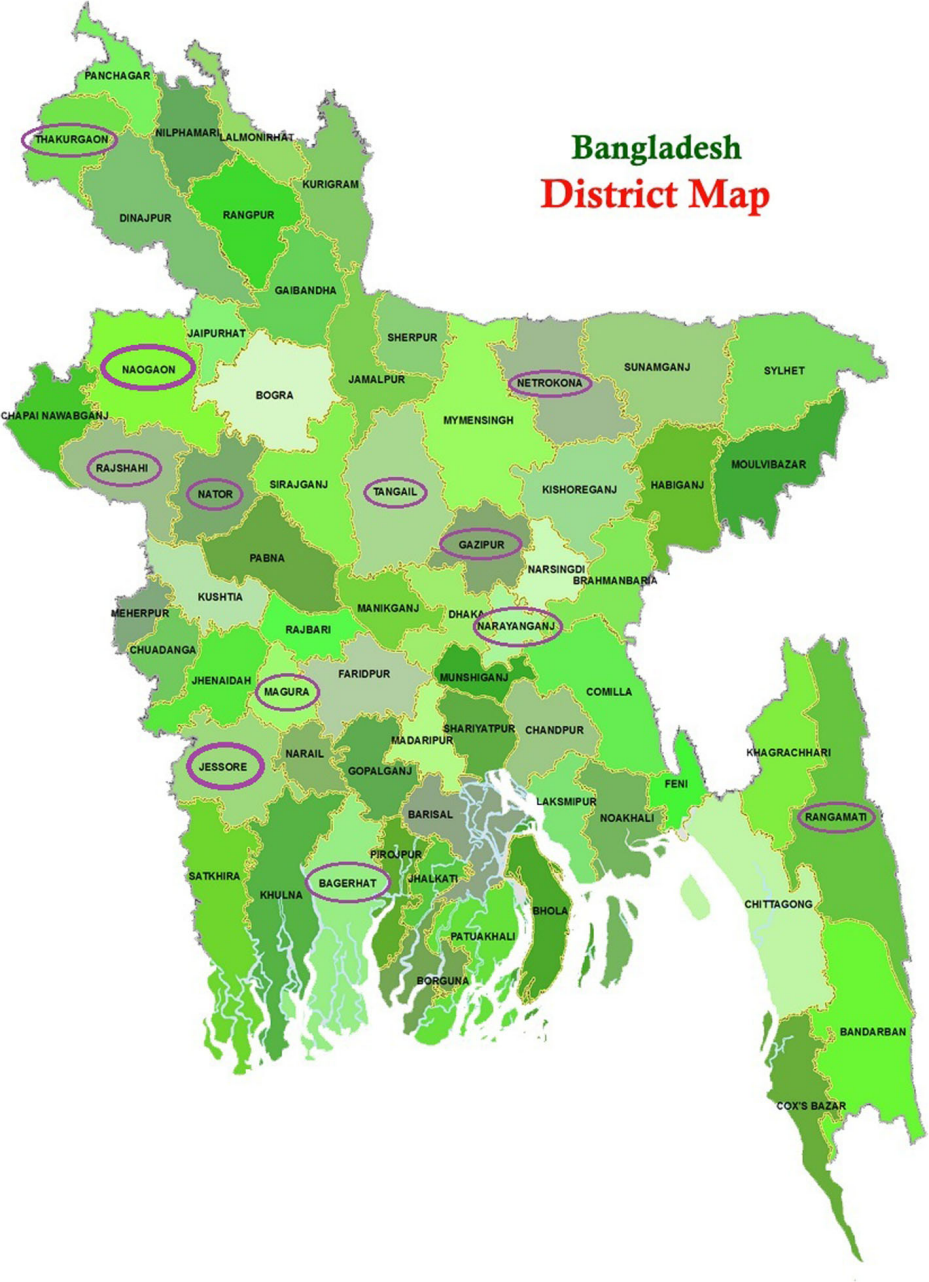
District map of Bangladesh. Circles showing the name of the informed districts from where the survey was conducted by authors. Map source: PNGWING (https://www.pngwing.com/en/free-png-hzazs)

It has been found that Bangladeshi FMPs from 12 different districts used *Cannabis sativa* to treat various ailments wherein the maximum number of cited ailment is sleep-associated problems (*n*=5), neuropsychiatric and CNS problems (*n*=5), and infections and respiratory problems (*n*=5) followed by rheumatism, gastrointestinal, gynecological (*n*=4 each), cancer, sexual, and other ailments (*n*=1 each) (see Table 1). Figure 1 shows the name of 12 districts of Bangladesh (Bagerhat, Gazipur, Jessore, Magura, Naogaon, Narayanganj, Natore, Netrakona, Rajshahi, Rangamati, Tangail and Thakurgaon) from where the ethnomedicinal plants’ information was surveyed.

**Table 1:** Number of cited ailments treated by folk medicine practitioners of Bangladesh using different parts of *Cannabis sativa*

Name of ailment	Number of citation (***n***=)	Parts used	Pharmacological justification (from literature)
Sleep problems (insomnia, to induce sleep, soporific)	5	Leaf, root, seed, inflorescence	Anticancer and antitumor (Lukhele and Motadi 2016; Velasco et al. 2012, 2016), analgesic (Argueta et al. 2020; Comelli et al. 2008), anti-arthritis (Lowin et al. 2020), anti-spasticity (Hagenbach et al. 2007; Leussink et al. 2012), antibacterial, antimicrobial (Appendino et al. 2008, Monika and Kaur, 2014, Pellegrini et al. 2020) Cardiovascular care (Garza-Cervantes et al., 2020) Digestive disorders (Goyal et al. 2017; Machado Rocha et al. 2008; Parker et al. 2011) Infection, wound injury (Ali et al. 2012; Sangiovanni et al. 2019) Hormonal effects (Brents 2016; Walker et al. 2019) Mental health (Khan et al. 2020) Headache/migraine (Leimuranta et al. 2018; Lochte et al. 2017), Neuroprotection (Fernández-Ruiz et al. 2015; Milano 2018) Sedative, insomnia (Shannon et al. 2019; Vigil et al. 2018)
Arthritis and pain (gout, rheumatism, cancer and arthritic pain)	4	Leaf, root, seed	
Gynecological disorders (dysmenorrhea, menorrhagia, expedite delivery)	4	Leaf, root	
Cancer	3	All parts, leaf, root, seed	
Sexual problems (erectile dysfunction, sex stimulation, low libido, pleasant sensation)	3	Leaf, root, seed	
Gastrointestinal problems (diarrhea, dyspepsia, strangulated hernia, poor digestion, dysentery)	4	Leaf, flower	
Neuropsychiatric and CNS (paralysis, psychosis, insanity)	5	Leaf, root	
Infections and respiratory problems (tetanus, wound, tuberculosis, cough, asthma)	5	Leaf, root, resin	
Other ailments (hypertension, headache, itch, increases bile secretion, abortifacient, dandruff, fever, urinary problems)	1	Leaf, root, resin

To the extent of our knowledge, there would be over 1000 ethnomedicinal plants’ survey reports on Bangladesh published. For this review study, over 200 ethnomedicinal plants survey articles were reviewed wherein 18 survey articles have mentioned *Cannabis sativa*. A total of 15 formulations have been identified from the 11 articles where the rest of the studied articles did not mention any mode of preparation.

The authors of the identified articles conducted the ethnobotanical field survey using open-ended and semi-structured questionnaire method. It is worth noting that there is at least one folk medicine practitioner in almost every village of Bangladesh. Most of the interviewed practitioners detailed in the identified articles are male by sex with an average age of 55–65 years, and they inherited treatment knowledge using medicinal plants from earlier generational people like a maternal grandfather. Mahnoor et al. (2015) have interviewed a 77-year-old female folk herbalist and Hasan et al. (2015) have also interviewed a female practitioner who was 55 years. Kona and Rahman (2016) have conducted ethnobotanical survey from December 2013 to June 2015 and interviewed a total of 156 people having age range 18–75 years old. Another ethnobotanical survey (Kadir et al. 2013) was conducted between January 2010 and June 2012 with an aim to interview at least six Kabiraj/Ayurved/Hakim/Unani practitioners in each area (Dhaka, Chittagong hill tracts, Rangamati, Bandarban, Cox’s bazaar, Mymensingh, Sylhet, Sundarbans, Rangpur, Rajshahi, and Barishal). A total of 1280 informants were interviewed with an age of 50–60 years old where major informants were male (67%) (Kadir et al. 2013). A tribal ethnobotanical survey was conducted in the two Santal communities residing in Thakurgaon district and reported to the use of Cannabis plant (Rahmatullah et al. 2009).

### Plant parts used and mode of preparation

*Cannabis sativa* leaf is the most frequently used plant part (53.8%) followed by root (23%), seed (7.7%), flower, inflorescence, resin, and all parts 3.8% respectively. FMPs generally follow simple preparation methods instead of a procedure of complex formulations. An oil-based preparation of Cannabis leaf is used to treat schizophrenia, and in severe conditions, leaves are used to make a vapor that is inhaled by the patient (Ahmed and Azam 2014). Leaf juice is taken orally for treating bloating, cough, and mucus (Rahmatullah et al. 2009), and leaf is also used to remove dandruff (Sultana and Rahman 2017). Macerated roots of *Cannabis sativa* and leaves of *Chromolaena odorata* are combined and taken for fever (Rahmatullah et al. 2011). One teaspoon powder of dry, crushed Cannabis leaf is added to water and taken once a day orally as a CNS depressant and to treat arthritis pain (Mawla et al. 2012). Root is made into a paste with 25 black pepper and given twice daily to treat insanity, tetanus, pain of dysmenorrhea, menorrhagia, and phthisis; seed oil is useful in rheumatism, cancer chemotherapy, and cancer pain (Siddique et al. 2006). The smoke of dried pistillate of flowering tops of Cannabis plant is passed through the rectum for relief from strangulated hernia and griping pains of dysentery (Kadir et al. 2013) (see Table 2).

**Table 2:** List of traditional uses of *Cannabis sativa* as phytomedicine used by the folk medicine practitioners of Bangladesh and potential therapeutic compounds

Ailment (s) treated	Part (s) used	Mode of preparations and administration	Reference	Potential therapeutic compounds (from literature)
Sedative, paralysis, narcotic	Leaf, root	Not given	Rahmatullah et al. 2010d	**Roots**: friedelan-3-one, epifriedelanol, β-sitosterol, ergost-5-en-3-ol, methyl hexadecanoate, pentadecanoic acid, 10E-hexadecenoic acid, 4-hydroxy-3-methoxybenzaldehyde, β-sitosterol-β-D-glucoside and p-coumaroyltyramine (Elhendawy et al. 2019) Triterpenoids (friedelin, epifriedelanol); alkaloids (cannabisativine, anhydrocannabisativine); carvone and dihydrocarvone; N-(p-hydroxy-β-phenylethyl)-p-hydroxy-trans-cinnamamide; sterols (sitosterol, campesterol, and stigmasterol) (Ryz et al. 2017)
Rheumatoid Arthritis	Leaf, root	Not given	Rahmatullah et al. 2010e	
Cancer, hypertension, antidote to poison, itch, rheumatoid arthritis.	Leaf, root	Not given	Rahmatullah et al. 2010f	
Bitter, increases bile secretion, hallucinogeni, sex stimulant, to induce sleep, to induce pleasant sensations, excessive menstruation, urination problems	Leaf	Not given	Rahmatullah et al. 2010g	
Fever	Root	Macerated roots of *Cannabis sativa* and leaves of *Chromolaena odorata* are combined and taken	Rahmatullah et al. 2011	**Leaves**: cannabispirketal, α-cannabispiranol 4’-O-β-D-glucopyranose, cannabispirenone-A, cannabispirone, 9,10-dihydro-2,3,5,6-tetramethoxyphenanthrene-1,4-dione, 4-hydroxy-2,3,6,7-tetramethoxy-9,10-dihydrophenanthrene, 4-hydroxy-2,3-dimethylnon-2-en-4-olide, Indole-2-carboxylic acid ethyl ester, cannflavin A, 6-geranylapigenin, 6-isopentenyl apigenin, cannflavin B, 8-isopentenyl isorhamnetin, orientin, vitexin, 4′-methoxy orientin, and cytisoside (Guo et al., 2017).
Bloating, cough, mucus.	Leaf	Leaf juice is taken orally for all three ailments.	Rahmatullah et al. 2009	
Wound infections of cattle	Leaf	Not given	Rashid et al. 2010	
CNS depressant, gout, arthritic pain	Leaf	One teaspoon powder obtained from crushed and dried leaf is added to water and taken once orally.	Mawla et al. 2012	
				**Seeds’ oil**: Cannabinoids (Cannabidiol, Δ9-tetrahydrocannabinol, Cannabiripsol, Cannabitriol, Cannabigerol, Cannabielsoin, Cannabinol, Cannabichromene, Cannabicitran) β-caryophyllene, myrcene, β-sitosterol, methyl salicylate, fatty acids (linoleic, α-linolenic, and oleic), campesterol, phytol, cycloartenol, and γ-tocopherol (Citti et al. 2019a; Leizer et al. 2000; Nabukenya et al. 2014)
Insomnia, coughs, low libido	Leaf, seed	Leaves and seeds are dried, powdered and made into balls of about 1/16 kg each. One ball is taken daily for coughs, mucus, as a narcotic and to induce sleep. The seeds are taken for sexual stimulation.	Nawaz et al. 2009	
Cancer	All parts	Not given	Mollik et al., 2010	
Insanity, tetanus, menstrual pain, tuberculosis, rheumatism, cancer chemotherapy, and cancer pain	Root, seed,	(a) Root is made into a paste with 25 black pepper and given twice daily for insanity and tetanus, also used for relieves pain of dysmenorrhea, menorrhagia, and phthisis (b) Seed oil is used in rheumatism, cancer chemotherapy and cancer pain.	Siddique et al. 2006	
Poor digestion, hallucinogenic, sexual dysfunction, insomnia, induce pleasant sensations, excessive menstruation, urination problems.	Leaf	Paste from leaves which has been heated	Walid et al. 2013	
Schizophrenia like psychotic problems	Leaf	(a) Leaves are used to make oil then massage on the scalp until cured. (b) If a patient is in severe condition, then the leaves are used to make vapor which is inhaled through the nose.	Ahmed and Azam 2014	
(a) Dandruff (b) Headache, asthma	Leaf, resin	(a) Leaves make a good snuff for deterging the brain; juice removes dandruff (b) The resin called Charas is used to prevent and cure headache and asthma.	Sultana and Rahman 2017	
Soporific, abortifacient	Leaf, Inflorescence	Not given	Kona and Rahman 2016	
(a) Strangulated hernia and griping pains of dysentery (b) Diarrhea, dyspepsia and bowel complaints.	Leaf, flower	(a) The smoke of dried pistillate of flowering tops which are coated with resinous exudation is passed through the rectum for relief from strangulated hernia and griping pains of dysentery (b) The preparation made specially from dried leaves and flowers known as bhang, siddhi or hashis is given to check diarrhea, dyspepsia and bowel complaints	Kadir et al., 2013	
Erectile dysfunction	Root	Root juice is orally taken	Hasan et al. 2015a	
To expedite delivery	Leaf	Leaves are fried in ghee and powdered and then orally taken with warm water.	Mahnoor et al., 2015	

## Discussion

We conducted an extensive study on combining detailed information on the traditional use of *Cannabis sativa* by folk medicine practitioners in Bangladesh. We corroborated ethnomedicinal usage of *Cannabis sativa* by pharmacological data. Our result indicates that different parts of the Cannabis plant were used by several folk medicine practitioners in Bangladesh to improve sleep quality, relief from pain, treating diseases, and psychological conditions, including gynecological diseases, sexual problems, gastrointestinal problems, various types of cancers, asthma, and respiratory problems, etc. (see Tables 1 and 2). Such a diverse set of cures and/or treatments have also been used by folk medicine practitioners and professionals around the world (Alsherbiny and Li 2018).

One of the most common use of Cannabis found by our current study is to reduce or relieve from pain (Kadir et al. 2013, Mawla et al. 2012, Siddique et al. 2006, Sultana and Rahman 2017). It is worth mentioning that in the USA, the National Academies of Sciences, Engineering, and Medicine has supported the use of Cannabis in the treatment of chronic pain in adults through their analysis of the current scientific evidence (Romero-Sandoval et al. 2018), and a team of researchers also (Xiaoxue et al. 2019) found strong evidence that Cannabis with moderate high levels of THC can significantly alleviate pain. Cannabinoids may be useful in treating rheumatoid arthritis due to their anti-inflammatory and immunomodulatory activity (Lowin et al. 2019, Sarzi-Puttini et al. 2019). Cannabis and cannabinoids have also potentials which showed promising results to alleviate pain related to rheumatic diseases (Gonen and Amital 2020, Haleem and Wright 2020). In the century between 1842 and 1942, Cannabis was part of Western pharmacopeias preferred to other preparations by physicians in migraine treatment (Russo 2001). It was also used to treat headache/migraine in Bangladesh (Sultana and Rahman 2017) by folk medicine practitioners. A cannabis-derived medicine containing THC and CBD, Nabiximols, is approved in several countries for pain and muscle spasticity (Überall 2020).

Another interesting finding of the current study is that Cannabis most likely has the ability to improve sleep quality, decrease sleep disturbances, and decrease sleep onset latency (Kuhathasan et al. 2019, Nawaz et al. 2009). These findings align with a double-blind clinical trial, which showed success in treating chronic insomnia by using Zelira’s proprietary cannabis formulation (ZTL-101) which was on target to launch in the Australian market by early Q3 2020 (Zelira Therapeutics 2020). Moreover, the use of cannabis to treat psychological conditions was also documented (Ahmed and Azam 2014). A small randomized clinical trial reported clinical improvement in patients with schizophrenia treated with CBD (Iseger and Bossong 2015), and nine ongoing clinical trials have registered which focused on the effects of CBD on psychotic disorders (Batalla et al. 2019).

Cannabis is potent in its gynecological actions, and cannabinoids might potentially influence the dysregulation of the endocannabinoid system whereby as specific agonists or antagonists (Luschnig and Schicho 2019, Russo 2002). FMPs of Bangladesh have used *Cannabis sativa* for gynecological problems like excessive menstrual and its pain, erectile dysfunctions (Hasan et al. 2015a, Rahmatullah et al. 2010g, Siddique et al. 2006, Walid et al. 2013). Similarly, herbal practitioners of Western Uganda and tribal communities near the Pak-Afghan border area also reported treating gynecological problems (abdominal pain, gonorrhea, sexual impotence, erectile dysfunctions) with the plant Cannabis (Aziz et al. 2018, Kamatenesi-Mugisha and Oryem-Origa 2005). The traditional usage of Cannabis was mentioned as an abortifacient in Bangladesh (Kona and Rahman 2006) together with Afghanistan, India, sand South Africa (Ross 2007). In animal studies, THC was found to impair women’s fertility (Misner and Favetta 2020, Wang et al., 2006), as well as Cannabis was reported to inhibit the capacity for male fertility (Payne et al. 2019). Despite this, Cannabis has been reported to be used as traditional aphrodisiac in Bangladesh (Nawaz et al. 2009, Rahmatullah et al., 2010g), India, and the USA (Ross 2007).

Cannabis was also used for traditional curative properties for asthma and respiratory problems in Bangladesh (Sultana and Rahman 2017), Cameroon (Noumi 2010), and South Africa (Ross 2007). Traditionally, Cannabis was also used to treat tuberculosis both in Bangladesh (Siddique et al. 2006) and South Africa (Lawal et al. 2014) as it exhibited antibacterial, antimicrobial properties (Appendino et al. 2008, Karas et al. 2020, Pellegrini et al. 2020). The contribution of cannabinoids in the treatment and management of gastrointestinal symptoms like nausea, vomiting, and visceral pain was found to be useful (Malik et al. 2015). The experimental data and robust preclinical evidence represent Cannabis and its compounds as a promising therapeutic in the treatment of intestinal inflammation and gastric mucosal lesions (Gyires and Zádori, 2016; Kienzl et al., 2020).

Cannabis was also mentioned for urination problems by FMPs of Bangladesh (Rahmatullah et al. 2010g, Walid et al. 2013). The 2004 clinical study discovered that Cannabis is effective which decreased urinary urgency, frequency, and urination at night in patients with advanced multiple sclerosis (Brady et al. 2004). Recent clinical trial studies showed the potentiality of cannabis medicine in improving lower urinary tract dysfunctions such as overactive bladder, interstitial cystitis/bladder pain syndrome, detrusor hyperreflexia, and urinary incontinence (Maniscalco et al. 2018, Nedumaran et al. 2017). Furthermore, Cannabis plant was also used by FMPs of Bangladesh for the treatment of various types of cancers (Mollik et al., 2010, Rahmatullah et al. 2010f). Hence, cannabinoids have great promising therapeutic potential for the treatment of various cancers (Kovalchuk and Kovalchuk 2020); in particular, CBD has reported vigorous anti-proliferative and pro-apoptotic effects on a wide variety of cancer types both in cultured cancer cell lines and in mouse tumor models. (Seltzer et al. 2020). Furthermore, traditional medicine practitioners in Zimbabwe used *Cannabis sativa* in cancer mainly for its analgesic, anti-nausea, and antiemetic properties (Matowa et al. 2020).

The endocannabinoid system and cannabinoids have started to get more and more considerable interests for therapeutic claims (Ryan et al. 2009) which could be observed by varieties of biological activities (Marcu 2016). Cannabis and cannabinoids can play the role of anti-nociceptive, anti-inflammatory, immunosuppressant anti-emetogenic activity, and anticonvulsant activity (Mensah and Adu-Gyamfi 2019). Cannabidiol (CBD) has been identified as therapeutic in human laboratory studies and clinical trials for epilepsy, anxiety, pain/inflammation, schizophrenia, various substance use disorders, post-traumatic stress disorder, and others (Sholler et al. 2020). Furthermore, the usage of Cannabis for therapeutic purposes reported not to increase risk of harm to self or others (Walsh et al. 2017), and the UN Commission on Narcotic Drugs already removed Cannabis and Cannabis resin from a category of the world's most dangerous substances (CND 2020; news.un.org/en/story/2020/12/1079132). Already, 33 states in the USA and several countries in the world have been using Cannabis for specific medical conditions (Sarma et al. 2020). In June 2018, the US Food and Drug Administration (FDA) approved the first cannabis-derived medicine, Epidiolex® (cannabidiol, CBD), for the treatment of seizure disorders (Abu-Sawwa et al. 2020).

However, Cannabis has been considered a narcotic drug, and its medicinal use ignored since the beginning of the twentieth century. It was the only recent decade that researchers started to conduct and follow up on the safe therapeutic potential of *Cannabis sativa*. It is worth mentioning that a search of the relevant scientific literatures provide support for the traditional usage of *Cannabis sativa* practiced by Bangladeshi folk medicine practitioners, thanks to the contribution of the cannabinoids to the unique biological properties.

## Conclusions

*Cannabis sativa* is a plant of phytochemical factories. The plant has been used for thousands of years around the world for its apparent biological activity and has not been reported being more toxic than several medicines in current clinical practice. Although illegal, in Bangladesh, folk medicine practitioners use Cannabis extensively to treat a large kind of ailments such as sleep problems (insomnia, induce sleep, soporific); arthritis and pain (gout, rheumatism, cancer, and arthritic pain); gynecological disorders (dysmenorrhea, menorrhagia, expedite delivery); sexual problems (erectile dysfunction, sex stimulation, low libido, pleasant sensation); gastrointestinal problems (diarrhea, dyspepsia, strangulated hernia, poor digestion, dysentery); neuropsychiatric and CNS (paralysis, psychosis, insanity); infections and respiratory problems (tetanus, wound, tuberculosis, cough, asthma); cancer; and other ailments including hypertension, headache, itch, increases bile secretion, abortifacient, dandruff, fever, and urinary problems.

Based on the literature review we performed, there is significant scientific evidence that provides support for the usage of *Cannabis sativa* as a traditional phytomedicine by folk medicine practitioners of Bangladesh. It is also a need to perform more biological evaluation towards a ray of hope establishing therapeutic guidelines of Cannabis and cannabinoids and to provide a strengthened pharmacological perspective about the prospects and challenges of Cannabis use in the future.

## Data Availability

Data sharing is not applicable to this article as no new data were generated or analyzed during this study.

## Abbreviations

CBD: Cannabidiol
CNS: Central nervous system
ECS: Endocannabinoid system
FMPs: Folk medicine practitioners
THC: (−)-*trans*-Δ^9^-Tetrahydrocannabinol
Δ9-THCP: Δ9-Tetrahydrocannabiphorol
PPV: Plant part value

## References

[CR1] Abu-Sawwa R, Scutt B, Park Y (2020). Emerging use of epidiolex (cannabidiol) in epilepsy. J Pediatr Pharmacol Ther.

[CR2] Ahmed MN, Azam MNK (2014). Traditional knowledge and formulations of medicinal plants used by the traditional medical practitioners of Bangladesh to treat schizophrenia like psychosis. Schizophr Res Treat.

[CR3] Ali EMM, Almagboul AZI, Khogali SME, Gergeir UMA (2012). Antimicrobial activity of Cannabis sativa L. Chin Med.

[CR4] Alsherbiny MA, Li CG (2018). Medicinal Cannabis-potential drug interactions. Medicines (Basel, Switzerland).

[CR5] Andre CM, Hausman JF, Guerriero G (2016). Cannabis sativa: the plant of the thousand and one molecules. Front Plant Sci.

[CR6] Appendino G, Gibbons S, Giana A, Pagani A, Grassi G, Stavri M, Smith (2008). Antibacterial cannabinoids from Cannabis sativa: a structure-activity study. J Nat Prod.

[CR7] Argueta DA, Ventura CM, Kiven S, Sagi V, Gupta K (2020). A balanced approach for cannabidiol use in chronic pain. Front Pharmacol.

[CR8] Azam MNK, Biswas S, Ahmed MN (2015). A cross-sectional study of ethnopharmacology in the Noakhali district of Bangladesh and exploring potential Ocular immunostimulatory activity of the medicinal plants for the treatment of Eye Infections. PharmacologyOnline.

[CR9] Azam MNK, Mannan MA, Ahmed MN (2014). Medicinal plants used by the traditional medical practitioners of Barendra and Shamatat (Rajshahi & Khulna Division) Region in Bangladesh for treatment of cardiovascular disorders. J Medicinal Plants Stud.

[CR10] Azam MNK, Rahman MM, Biswas S, Ahmed MN (2016). Appraisals of Bangladeshi medicinal plants used by folk medicine practitioners in the prevention and management of malignant neoplastic diseases. Int Sch Res Notices.

[CR11] Aziz MA, Khan AH, Ullah H, Adnan M, Hashem A, Abd Allah EF (2018). Traditional phytomedicines for gynecological problems used by tribal communities of Mohmand Agency near the Pak-Afghan border area. Rev Bras Farmacogn.

[CR12] Barrales-Cureño HJ, López-Valdez LG, Reyes C, Cetina-Alcalá VM, Vasquez-García I, Diaz-Lira OF, Herrera-Cabrera BE (2020). Chemical characteristics, therapeutic uses, and legal aspects of the cannabinoids of Cannabis sativa: a review. Braz Arch Biol Technol.

[CR13] Batalla A, Janssen H, Gangadin SS, Bossong MG (2019). The potential of cannabidiol as a treatment for psychosis and addiction: who benefits most? A systematic review. J Clin Med.

[CR14] Biswas KR, Khan T, Monalisa MN, Swarna A, Ishika T, Rahman M, Rahmatullah M (2011). Medicinal plants used by folk medicinal practitioners of four adjoining villages of Narail and Jessore Districts, Bangladesh. Am-Eursion J Sustain Agric.

[CR15] Bonini SA, Premoli M, Tambaro S, Kumar A, Maccarinelli G, Memo M, Mastinu A (2018). Cannabis sativa. A comprehensive ethnopharmacological review of a medicinal plant with a long history. J Ethnopharmacol.

[CR16] Booth JK, Bohlmann J (2019). Terpenes in Cannabis sativa - from plant genome to humans. Plant Sci.

[CR17] Brady CM, DasGupta R, Dalton C, Wiseman OJ, Berkley KJ, Fowler CJ (2004). An open-label pilot study of cannabis-based extracts for bladder dysfunction in advanced multiple sclerosis. Multiple sclerosis (Houndmills, Basingstoke, England).

[CR18] Brenneisen R, ElSohly MA (2007). Chemistry and analysis of phytocannabinoids and other Cannabis constituents. Marijuana and the Cannabinoids. Forensic Science And Medicine.

[CR19] Brents LK (2016). Marijuana, the endocannabinoid system and the female reproductive system. Yale J Biol Med.

[CR20] Burstein S (1997). Marijuana as a medicine. Nature.

[CR21] Calvi L, Pavlović R, Panseri S, Giupponi L, Leoni V, Giorgi A (2018). Quality traits of medical Cannabis sativa L. inflorescences and derived products based on comprehensive mass-spectrometry analytical investigation. Recent Advances in Cannabinoid Research.

[CR22] Canada H (2018). Information for health care professionals: Cannabis (marihuana, marihuana) and the cannabinoids.

[CR23] Chattopadhyaya U (2018) Naogaon and the world: intoxication, commoditisation, and imperialism in South Asia and the Indian Ocean 1840–1940 (PhD thesis, University of Illinois).

[CR24] Citti C, Linciano P, Panseri S, Vezzalini F, Forni F, Vandelli MA, Cannazza G (2019). Cannabinoid profiling of hemp seed oil by liquid chromatography coupled to high-resolution mass spectrometry. Front Plant Sci.

[CR25] Citti C, Linciano P, Russo F, Luongo L, Iannotta M, Maione S, Laganà A, Capriotti AL, Forni F, Vandelli MA, Gigli G, Cannazza G. A novel phytocannabinoid isolated from Cannabis sativa L. with an in vivo cannabimimetic activity higher than Δ9-tetrahydrocannabinol: Δ9-Tetrahydrocannabiphorol. 2019;9(1):Sci Rep, 20335. 10.1038/s41598-019-56785-1. 10.1038/s41598-019-56785-1 PMC6937300 31889124

[CR26] Clarke RC, Merlin M (2013). Cannabis: evolution and ethnobotany.

[CR27] Comelli F, Giagnoni G, Bettoni I, Colleoni M, & Costa B (2008) Antihyperalgesic effect of a Cannabis sativa extract in a rat model of neuropathic pain: mechanisms involved. Phytotherapy research : PTR 22(8):1017–1024. https://doi.org/10.1002/ptr.2401 10.1002/ptr.2401 18618522

[CR28] Corroon J, Phillips JA (2018). A cross-sectional study of cannabidiol users. Cannabis Cannabinoid Res.

[CR29] Cota D, Marsicano G, Luts B, Vicennati V, Stalla GK, Pasquali R, Pagotto U (2003). Endogenous cannabinoid system as a modulator of food intake. Int J Obes Relat Metab Disord.

[CR30] Elhendawy MA, Wanas AS, Radwan MM, Azzaz NA, Toson ES, ElSohly MA (2019). Chemical and biological studies of Cannabis sativa roots. Med Cannabis Cannabinoids.

[CR31] Faruque MO, Uddin SB, Barlow JW, Hu S, Dong S, Cai Q, Li X, Hu X (2018). Quantitative ethnobotany of medicinal plants used by indigenous communities in the Bandarban District of Bangladesh. Front Pharmacol.

[CR32] Fernández-Ruiz J, Moro MA, Martínez-Orgado J (2015). Cannabinoids in neurodegenerative disorders and stroke/brain trauma: from preclinical models to clinical applications. Neurotherapeutics.

[CR33] Garza-Cervantes JA, Ramos-González M, Lozano O, Jerjes-Sánchez C, García-Rivas G (2020). Therapeutic applications of cannabinoids in cardiomyopathy and heart failure. Oxid Med Cell Longev.

[CR34] Gomez-Beloz A (2002). Plant use knowledge of the Winikina Warao: The case for questionnaires in ethnobotany. Econ Botany.

[CR35] Gonen T & Amital H (2020) Cannabis and cannabinoids in the treatment of rheumatic diseases. Rambam Maimonides medical journal, 11(1), e0007. https://doi.org/10.5041/RMMJ.10389 10.5041/RMMJ.10389 PMC7000161 32017684

[CR36] Goyal H, Singla U, Gupta U, May E (2017). Role of cannabis in digestive disorders. Eur J Gastroenterol Hepatol.

[CR37] Guo TT, Zhang JC, Zhang H, Liu QC, Zhao Y, Hou YF, Bai L, Zhang L, Liu XY LXQ, Zhang SY, Bai NS (2017). Bioactive spirans and other constituents from the leaves of Cannabis sativa f. sativa. J Asian Nat Prod Res.

[CR38] Gyires K, Zádori ZS (2016). Role of cannabinoids in gastrointestinal mucosal defense and inflammation. Curr Neuropharmacol.

[CR39] Hagenbach U, Luz S, Ghafoor N, Berger JM, Grotenhermen F, Brenneisen R, Mäder M (2007). The treatment of spasticity with Δ9-tetrahydrocannabinol in persons with spinal cord injury. Spinal Cord.

[CR40] Haleem R & Wright R (2020). A scoping review on clinical trials of pain reduction with Cannabis administration in adults. J Clin Med Res, 12(6), 344–351. https://doi.org/10.14740/jocmr4210 10.14740/jocmr4210 PMC7295551 32587650

[CR41] Haque M (2020). History of Bangladesh Rock, The Legacy of Azam Khan.

[CR42] Haque MI (1993). Madakashakti: Jatio o vishshya poriprekshit [Drug addiction: National and world perspective].

[CR43] Hasan MM, Rahman MA, Amin R, Akter MH, Norin N, Nargis T, Das PR, Islam MT, Hossan MS, Rahmatullah M (2015). Do folk medicinal plants have scientific validity behind their uses? Some notes on plants used in Jessore District, Bangladesh. World J Pharm Pharm Sci.

[CR44] Hasan MN, Ahmed MN, Bhuiyan MZA, Rahman MM, Azam MNK, Rahmatullah M (2012) Medicinal plants used in treatment of tumors: results from a survey of folk medicinal practitioners. In: An International Conference on Green Chemistry for Sustainable Development, Jessore Science & Technology University, Bangladesh, 14 July 2012

[CR45] Hasan MN, Azam MNK, Ahmed MN, Hirashima A (2015). A randomized ethnomedicinal survey of snakebite treatment in southwestern parts of Bangladesh. J Tradit Complement Med.

[CR46] Hossan MS, Hanif A, Agarwala B, Sarwar MS, Karim M, Taufiq-ur-Rahman M, Jahan M, Rahmatullah M (2010). Traditional use of medicinal plants in Bangladesh to treat urinary tract infections and sexually transmitted diseases. Ethnobotany Res Appl.

[CR47] Iseger TA, Bossong MG (2015). A systematic review of the antipsychotic properties of cannabidiol in humans. Schizophr Res.

[CR48] Jahan FI, Hasan MRU, Jahan R, Seraj S, Chowdhury AR, Islam MT (2011). A comparison of medicinal plant usage by folk medicinal practitioners of two adjoining villages in Lalmonirhat District, Bangladesh. Am-Eurasian J Sustain Agric.

[CR49] Jin D, Dai K, Xie Z, Chen J (2020). Secondary metabolites profiled in Cannabis inflorescences, leaves, stem barks, and roots for medicinal purposes. Sci Rep.

[CR50] Kadir MF, Bin Sayeed MS, Mia MM (2013). Ethnopharmacological survey of medicinal plants used by traditional healers in Bangladesh for gastrointestinal disorders. J Ethnopharmacol.

[CR51] Kala CP, Farooquee NA, Dhar U (2004). Prioritization of medicinal plants on the basis of available knowledge, existing practices and use value status in Uttaranchal. India Biodiv Conser.

[CR52] Kamatenesi-Mugisha M, Oryem-Origa H (2005). Traditional herbal remedies used in the management of sexual impotence and erectile dysfunction in western Uganda. African Health Sciences.

[CR53] Karas JA, Wong LJM, Paulin OKA, Mazeh AC, Hussein MH, Li J, Velkov T (2020). The antimicrobial activity of Cannabinoids. Antibiotics (Basel).

[CR54] Khan R, Naveed S, Mian N, Fida A, Raafey MA, Aedma KK (2020). The therapeutic role of cannabidiol in mental health: a systematic review. J Cannabis Res.

[CR55] Kienzl M, Storr M, Schicho R (2020). Cannabinoids and opioids in the treatment of inflammatory bowel diseases. Clin Transl Gastroenterol.

[CR56] Kona S, Rahman AM (2016). Inventory of medicinal plants at Mahadebpur Upazila of Naogaon District, Bangladesh. Appl Ecol Environ Sci.

[CR57] Kovalchuk O, Kovalchuk I (2020). Cannabinoids as anticancer therapeutic agents. Cell Cycle.

[CR58] Kuhathasan N, Dufort A, MacKillop J, Gottschalk R, Minuzzi L, Frey BN (2019). The use of cannabinoids for sleep: a critical review on clinical trials. Exp Clin Psychopharmacol.

[CR59] Lash R (2010). Industrial Hemp: the crop for the seventh generation. Am Indian Law Rev.

[CR60] Lawal IO, Grierson DS, Afolayan AJ (2014). Phytotherapeutic information on plants used for the treatment of tuberculosis in Eastern Cape Province, South Africa. Evid Based Complement Alternat Med.

[CR61] Leimuranta P, Khiroug L, Giniatullin R (2018). Emerging role of (endo)cannabinoids in migraine. Front Pharmacol.

[CR62] Leizer C, Ribnicky D, Poulev A, Dushenkov S, Raskin I (2000). The composition of hemp seed oil and its potential as an important source of nutrition. J Nutraceuticals Funct Med Foods.

[CR63] Leussink VI, Husseini L, Warnke C, Broussalis E, Hartung HP, Kieseier BC (2012). Symptomatic therapy in multiple sclerosis: the role of cannabinoids in treating spasticity. Therapeutic Adv Neurol Disord.

[CR64] Lochte BC, Beletsky A, Samuel NK, Grant I (2017). The use of Cannabis for headache disorders. Cannabis Cannabinoid Res.

[CR65] Lowin T, Schneider M, Pongratz G (2019). Joints for joints: cannabinoids in the treatment of rheumatoid arthritis. Curr Opin Rheumatol.

[CR66] Lowin T, Tingting R, Zurmahr J, Classen T, Schneider M, Pongratz G (2020). Cannabidiol (CBD): a killer for inflammatory rheumatoid arthritis synovial fibroblasts. Cell Death Dis.

[CR67] Lozano I (2001). The therapeutic use of Cannabis sativa (L.) in Arabic medicine. J Cannabis Ther.

[CR68] Lukhele ST, Motadi LR (2016). Cannabidiol rather than Cannabis sativa extracts inhibit cell growth and induce apoptosis in cervical cancer cells. BMC complementary and alternative medicine.

[CR69] Luschnig P, Schicho R (2019). Cannabinoids in gynecological diseases. Med Cannabis Cannabinoids.

[CR70] Machado Rocha FC, Stéfano SC, De Cássia HR, Rosa Oliveira LM, Da Silveira DX (2008). Therapeutic use of Cannabis sativa on chemotherapy-induced nausea and vomiting among cancer patients: systematic review and meta-analysis. Eur J Cancer Care.

[CR71] Mahmud H (2008). Drug addict ion and identity politics: the spiritual use of ganja in Bangladesh. Contemp Justice Rev.

[CR72] Mahnoor N, Moonmoon IF, Saha T, Mahamud K, Biswas s, Islam E, Rahmatullah M (2015) Medicinal plants of a folk herbalist in Tangail District, Bangladesh. Am-Eurasian J Sustain Agric 9(4):74-82.

[CR73] Malek MA (1999). Madokashokti: Oitihasik prekkhapot [Drug addiction: Historical background].

[CR74] Malik Z, Baik D, Schey R (2015). The role of cannabinoids in regulation of nausea and vomiting, and visceral pain. Curr Gastroenterol Rep.

[CR75] Maniscalco GT, Aponte R, Bruzzese D, Guarcello G, Manzo V, Napolitano M, Moreggia O, Chiariello F, Florio C. THC/CBD oromucosal spray in patients with multiple sclerosis overactive bladder: a pilot prospective study. Neurolog Sci. 2018;39(1):97–102. 10.1007/s10072-017-3148-6. 10.1007/s10072-017-3148-6 29052091

[CR76] Marcu JP, Victor RP (2016). Chapter 62 - An overview of major and minor phytocannabinoids. Neuropathology of Drug Addictions and Substance Misuse.

[CR77] Matowa PR, Gundidza M, Gwanzura L, Nhachi CFB (2020). A survey of ethnomedicinal plants used to treat cancer by traditional medicine practitioners in Zimbabwe. BMC Complement Med Ther.

[CR78] Mawla F, Khatoon S, Tehana F, Jahan S, Shelly MMR, Hossain S, Haq WM, Rahman S, Debnath K, Rahmatullah M (2012). Ethnomedicinal plants of folk medicinal practitioners in four villages of Natore and Rajshahi districts, Bangladesh. Am-Eurasian J Sustain Agric.

[CR79] Mensah KB, Adu-Gyamfi PKT (2019). To legalize cannabis in Ghana or not to legalize? Reviewing the pharmacological evidence. Arch Pharm Pharma Sci.

[CR80] Milano W. Neuroprotection by cannabinoids in neurodegenerative diseases. Alzheimers Dement Cogn Neurol. 2018. 10.15761/ADCN.1000120.

[CR81] Misner M and Favetta L (2020). Effects of delta-9-tetrahydrocannabinol (THC) on oocyte competence and early embryonic development. J Endocr Soc 4(Issue Supplement_1)0, MON-010. 10.1210/jendso/bvaa046.507. 10.3389/ftox.2021.647918 PMC8915882 35295104

[CR82] Mollik AH, Hossan S, Jahan R, Rahmatullah M. Some medicinal plants used in Bangladesh in traditional medicinal treatment of various forms of cancer. Planta Medica. 2009:75–PE77. 10.1055/s-0029-1234638.

[CR83] Mollik MAH, McField R, Faruque MR, Thapa KK, Hassan AI and Ahmmed B (2010) .Ethnomedicinal uses of some medicinal plants for prevention against all forms of cancer by the traditional healers in Gazipur district of Bangladesh. In: AACR International conference on frontiers in cancer prevention research. Cancer Prev Res Vol 3

[CR84] Monika KN, Kaur M (2014). Antimicrobial analysis of leaves of Cannabis sativa. J Sci.

[CR85] Nabukenya I, Rubaire-Akiiki C, Olila D, Ikwap K, Höglund J (2014). Ethnopharmacological practices by livestock farmers in Uganda: survey experiences from Mpigi and Gulu districts. J Ethnobiol Ethnomed.

[CR86] Nawaz AHM, Hossain M, Karim M, Khan M, Jahan R, Rahmatullah M (2009). An ethnobotanical survey of Rajshahi district in Rajshahi division, Bangladesh. Am-Eurasian J Sustain Agric.

[CR87] Nedumaran B, Rudra P, Gaydos J, Kumar S, Meacham RB, Burnham EL, Malykhina AP (2017). Impact of regular Cannabis use on biomarkers of lower urinary tract function. Urology.

[CR88] Noumi E (2010). Ethno-medico-botanical survey of medicinal plants in the treatment of asthma in the Nkongsamba Region, Cameroon. Indian J Tradit Knolwedge.

[CR89] Parker LA, Rock EM, Limebeer CL (2011). Regulation of nausea and vomiting by cannabinoids. Br J Pharmacol.

[CR90] Payne KS, Mazur DJ, Hotaling JM, Pastuszak AW (2019). Cannabis and male fertility: a systematic review. J Urol.

[CR91] Pellegrini M, Palmieri S, Ricci A, Serio A, Paparella A, Lo Sterzo C. In vitro antioxidant and antimicrobial activity of Cannabis sativa L. cv 'Futura 75' essential oil. Nat Prod Res. 2020:1–5. 10.1080/14786419.2020.1813139. 10.1080/14786419.2020.1813139 32865042

[CR92] Quinlan MB (2011) Ethnomedicine. In A companion to medical anthropology (eds M. Singer and P.I. Erickson). doi:10.1002/9781444395303.ch19

[CR93] Radwan MM, Elsohly MA, Slade D, Ahmed SA, Khan IA, Ross SA (2009). Biologically active cannabinoids from high-potency Cannabis sativa. J Nat Prod.

[CR94] Rahmatullah M, Azam MN, Khatun Z, Seraj S, Islam F, Rahman MA, Jahan S, Aziz MS (2012). Medicinal plants used for treatment of diabetes by the Marakh sect of the Garo tribe living in Mymensingh district, Bangladesh. Afr J Tradit Complement Altern Med.

[CR95] Rahmatullah M, Azam MNK, Rahman MM, Seraj S, Mahal MJ, Mou SM, Nasrin D, Khatun Z, Islam F, Chowdhury MH (2011). A survey of medicinal plants used by Garo and non-Garo traditional medicinal practitioners in two villages of Tangail District, Bangladesh. Am-Eurasian J Sustain Agric.

[CR96] Rahmatullah M, Hossan S, Khatun A, Seraj S, Jahan R (2012). Medicinal plants used by various tribes of Bangladesh for treatment of malaria. Malar Res Treat.

[CR97] Rahmatullah M, Jahan R, Azad AK, Seraj S, Rahman M, Chowdhury AR, Begum R, Nasrin D, Khatun Z, Hossain MS, Khatun A, Miajee ZUME (2010). A randomized survey of medicinal plants used by folk medicinal practitioners in six districts of Bangladesh to treat rheumatoid arthritis. Adv in Nat Appl Sci.

[CR98] Rahmatullah M, Jahan R, Azad AK, Seraj S, Rahman MM, Chowdhury AR, Begum R, Nasrin D, Khatun Z, Hossain MS, Khatun MA, Miajee ZUME, Jahan FI (2010). A randomized survey of medicinal plants used by folk medicinal practitioners in ten districts of Bangladesh to treat leprosy. Adv in Nat Appl Sci.

[CR99] Rahmatullah M, Jahan R, Azak AK, Seraj S, Rahman MM, Chowdhury AR, Begum R, Nasrin D, Khatun Z, Hossain MS, Khatun A, Miajee ZUME (2010). Medicinal plants used by folk medicinal practitioners in three villages of Natore and Rajshahi districts, Bangladesh. Am-Eurasian J Sustain Agric.

[CR100] Rahmatullah M, Jahan R, Hossan MS, Seraj S, Rahman MM, Chowdhury AR, Begum R, Nasrin D, Khatun Z, Hossain MS, Khatun MA, Jahan FI (2010). A comparative analysis of medicinal plants used by three tribes of Chittagong hill tracts region, Bangladesh to treat leukorrhea. Adv in Nat Appl Sci.

[CR101] Rahmatullah M, Jahan R, Hossan MS, Seraj S, Rahman MM, Chowdhury AR, Miajee ZUME, Nasrin D, Khatun Z, Jahan FI, Khatun MA (2010). A comparative analysis of medicinal plants used by several tribes of Chittagong hill tracts region, Bangladesh to treat helminthic infections. Adv in Nat Appl Sci.

[CR102] Rahmatullah M, Jahan R, Rahman MM, Seraj S, Nasrin D, Khatun Z (2010). A survey of medicinal plants used by folk medicinal practitioners for treatment of gastrointestinal disorders in randomly selected areas of four districts of Bangladesh. Adv Nat Appl Sci.

[CR103] Rahmatullah M, Mollik MA, Islam M, Islam MR, Jahan F, Khatun Z, Seraj S, Chowdhury MH, Islam F, Miajee ZU, Jahan R (2010). A survey of medicinal and functional food plants used by the folk medicinal practitioners of three villages in Sreepur Upazilla, Magura district, Bangladesh. Am-Eurasian J Sustain Agric.

[CR104] Rahmatullah M, Mollik MAH, Azam ATMA, Islam MR, Chowdhury MAS, Jahan R, Chowdhury MH, Rahman T (2009). Ethnobotanical survey of the Santal tribe residing in Thakurgaon District, Bangladesh. Am-Eurasian J Sustain Agric.

[CR105] Rahmatullah M, Mollik MAH, Khatun MA, Jahan R, Chwdhury AR, Seraj S, Hossain MS, Nasrin D, Khatun Z (2010). A survey on the use of medicinal plants by folk medicinal practitioners in five villages of Boalia Sub-district, Rajshahi District, Bangladesh. Adv in Nat Appl Sci.

[CR106] Rashid MH, Tanzin R, Ghosh KC, Jahan R, Khaun MA, rahmatullah M. (2010). An ethnoveterinary survey of medicinal plants used to treat cattle diseases in Birishiri area, Netrakona district, Bangladesh. Adv in Nat Appl Sci.

[CR107] Rashid S (2017) Heritage, Folk Medicine and Kaviraji treatment in Bangladesh. In: Eivind F (ed) Traditional Medicine: sharing experiences from the field, Chapter 5, ICHCAP.

[CR108] Report of the Bengal Provincial Banking Enquiry Committee, 1929-30. Vol. I; Calcutta: Government Press, 1930; 313-314.

[CR109] Romero-Sandoval EA, Fincham JE, Kolano AL, Sharpe B, Alvarado-Vazquez PA (2018). Cannabis for chronic pain: challenges and considerations. Pharmacotherapy.

[CR110] Ross IA. Medicinal plants of the world, Volume 3: Chemical constituents, traditional and modern medicinal uses. Springer Sci Bus Media. 2007. 10.1007/978-1-59259-887-8.

[CR111] Russo EB (2001). Hemp for headache: an in-depth historical and scientific review of cannabis in migraine treatment. J Cannabis Ther.

[CR112] Russo EB (2002). Cannabis treatments in obstetrics and gynecology: a historical review. J Cannabis Ther.

[CR113] Russo EB, Marcu J (2017). Cannabis pharmacology: the usual suspects and a few promising leads. Adv Pharmacol.

[CR114] Ryan D, Drysdale AJ, Lafourcade C, Pertwee RG, Platt B (2009). Cannabidiol targets mitochondria to regulate intracellular Ca^2+^ levels. J Neurosci.

[CR115] Ryz NR, Remillard DJ, Russo EB (2017). Cannabis roots: a traditional therapy with future potential for treating inflammation and pain. Cannabis and cannabinoid research.

[CR116] Sangiovanni E, Fumagalli M, Pacchetti B, Piazza S, Magnavacca A, Khalilpour S, Melzi G, Martinelli G, Dell'Agli M (2019). Cannabis sativa L. extract and cannabidiol inhibit in vitro mediators of skin inflammation and wound injury. Phytother Res.

[CR117] Šantić Ž, Pravdić N, Bevanda M, Galić K (2017). The historical use of medicinal plants in traditional and scientific medicine. Psychiatria Danubina.

[CR118] Sarma ND, Waye A, ElSohly MA, Brown PN, Elzinga S, Johnson HE, Marles RJ, Melanson JE, Russo E, Deyton L, Hudalla C, Vrdoljak GA, Wurzer JH, Khan IA, Kim NC, Giancaspro GI (2020). Cannabis inflorescence for medical purposes: USP considerations for quality attributes. J Nat Prod.

[CR119] Sarzi-Puttini P, Ablin J, Trabelsi A, Fitzcharles MA, Marotto D, Häuser W (2019). Cannabinoids in the treatment of rheumatic diseases: Pros and cons. Autoimmun Rev.

[CR120] Seltzer ES, Watters AK, MacKenzie D, Jr Granat LM, Zhang D (2020). Cannabidiol (CBD) as a promising anti-cancer drug. Cancers.

[CR121] Shannon S, Lewis N, Lee H, Hughes S (2019). Cannabidiol in anxiety and sleep: a large case series. Perm J.

[CR122] Sholler DJ, Schoene L, Spindle TR (2020). Therapeutic efficacy of cannabidiol (CBD): a review of the evidence from clinical trials and human laboratory studies. Curr Addict Rep.

[CR123] Siddique NA, Jaime A, da Silva T, Bari MA (2006). Preservation of indigenous knowledge regarding important and endangered medicinal plants in Rajshahi District of Bangladesh. J Plant Sci.

[CR124] Singh B, Singh B, Kishor A, Singh S, Bhat MN, Surmal O, Musarella CM (2020). Exploring plant-based ethnomedicine and quantitative ethnopharmacology: medicinal plants utilized by the population of Jasrota Hill in Western Himalaya. Sustainability.

[CR125] Small E, Chandra S, Lata H, ElSohly M (2017). Classification of Cannabis sativa L. in Relation to agricultural, biotechnological, medical and recreational utilization. Cannabis sativa L. - Botany and Biotechnology.

[CR126] Souza E, Williamson EM, & Hawkins JA (2018) Which plants used in ethnomedicine are characterized? Phylogenetic patterns in traditional use related to research effort. Front Plant Sci, 9, 834. https://doi.org/10.3389/fpls.2018.00834 10.3389/fpls.2018.00834 PMC6019821 29973942

[CR127] Sultana R, Rahman AHMM (2017). Documentation of medicinal plants at the village Kholabaria of Natore District, Bangladesh. Acad J Life Sci.

[CR128] Turner SE, Williams CM, Iversen L, Whalley BJ (2017). Molecular pharmacology of phytocannabinoids. Prog Chem Org Nat Prod.

[CR129] Überall MA (2020). A review of scientific evidence for THC: CBD oromucosal spray (nabiximols) in the management of chronic pain. J Pain Res.

[CR130] Velasco G, Hernández-Tiedra S, Dávila D, Lorente M (2016). The use of cannabinoids as anticancer agents. Prog Neuropsychopharmacol Biolog Psychiatry.

[CR131] Velasco G, Sanchez C, Guzman M (2012). Towards the use of cannabinoids as antitumour agents. Nat Rev Cancer.

[CR132] Vigil JM, Stith SS, Diviant JP, Brockelman F, Keeling K, Hall B. Effectiveness of raw, natural medical Cannabis flower for treating insomnia under naturalistic conditions. Medicines (Basel, Switzerland). 2018;5(3):–75. 10.3390/medicines5030075. 10.3390/medicines5030075 PMC6164964 29997343

[CR133] Walid R, Suvro KFA, Harun-or-Rashid M, Mukti M, Rahman S, Rahmatullah M (2013). Ethnomedicinal plants of folk medicinal practitioners of two villages in Bagerhat district of Bangladesh. Am-Eurasian J Sustain Agric.

[CR134] Walker OS, Holloway AC, Raha S (2019). The role of the endocannabinoid system in female reproductive tissues. J Ovarian Res.

[CR135] Walsh Z, Gonzalez R, Crosby KS, Thiessen M, Carroll C, Bonn-Miller MO (2017). Medical cannabis and mental health: a guided systematic review. Clin Psychol Rev.

[CR136] Wang H, Xie H, Guo Y, Zhang H, Takahashi T, Kingsley PJ, Marnett LJ, Das SK, Cravatt BF, Dey SK (2006). Fatty acid amide hydrolase deficiency limits early pregnancy events. J Clin Invest.

[CR137] Zelira Therapeutics (2020) Zelira Therapeutics meets primary endpoints for phase (1b/2a) medicinal cannabis trial for insomnia. https://www.asx.com.au/asxpdf/20200407/pdf/44gs3s7427zrmt.pdf. Accessed 9 Feb 2021.

[CR138] Zuardi AW (2006). History of cannabis as a medicine: a review. Braz J Psychiatry.

[CR139] Zwenger SR (2014). The biotechnology of *Cannabis sativa*.

